# Integrative Analyses of miRNA-mRNA Interactions Reveal let-7b, miR-128 and MAPK Pathway Involvement in Muscle Mass Loss in Sex-Linked Dwarf Chickens

**DOI:** 10.3390/ijms17030276

**Published:** 2016-02-24

**Authors:** Wen Luo, Shumao Lin, Guihuan Li, Qinghua Nie, Xiquan Zhang

**Affiliations:** 1 Department of Animal Genetics, Breeding and Reproduction, College of Animal Science, South China Agricultural University, Guangzhou 510642, Guangdong, China; lw729@stu.scau.edu.cn (W.L.); guihuanli@stu.scau.edu.cn (G.L.); nqinghua@scau.edu.cn (Q.N.); 2 Guangdong Provincial Key Lab of Agro-Animal Genomics and Molecular Breeding, South China Agricultural University, Guangzhou 510642, Guangdong, China; 3 Key Lab of Chicken Genetics, Breeding and Reproduction, Ministry of Agriculture, South China Agricultural University, Guangzhou 510642, Guangdong, China; 4 College of Life Science, Foshan University, Foshan 528231, Guangdong, China; shumaolin@stu.scau.edu.cn

**Keywords:** sex-link dwarf chicken, growth hormone receptor, network, microRNA, muscle development

## Abstract

The sex-linked dwarf (SLD) chicken is an ideal model system for understanding growth hormone (GH)-action and growth hormone receptor (GHR) function because of its recessive mutation in the *GHR* gene. Skeletal muscle mass is reduced in the SLD chicken with a smaller muscle fiber diameter. Our previous study has presented the mRNA and miRNA expression profiles of the SLD chicken and normal chicken between embryo day 14 and seven weeks of age. However, the molecular mechanism of *GHR*-deficient induced muscle mass loss is still unclear, and the key molecules and pathways underlying the *GHR*-deficient induced muscle mass loss also remain to be illustrated. Here, by functional network analysis of the differentially expressed miRNAs and mRNAs between the SLD and normal chickens, we revealed that let-7b, miR-128 and the MAPK pathway might play key roles in the *GHR*-deficient induced muscle mass loss, and that the reduced cell division and growth are potential cellular processes during the SLD chicken skeletal muscle development. Additionally, we also found some genes and miRNAs involved in chicken skeletal muscle development, through the MAPK, PI3K-Akt, Wnt and Insulin signaling pathways. This study provides new insights into the molecular mechanism underlying muscle mass loss in the SLD chickens, and some regulatory networks that are crucial for chicken skeletal muscle development.

## 1. Introduction

The sex-linked dwarf (SLD) chicken is caused by a recessive mutation of the growth hormone receptor (*GHR*) gene located on the Z chromosome. This mutation results in a number of phenotypic and physiological alterations of the SLD chickens. For example, shorter shanks, lower basal metabolism, higher serum growth hormone (GH), lower serum insulin-like growth factor-1 (IGF-1), higher tolerance to heat and higher feed conversion rate of SLD chickens are observed compared to those of normal chickens [1,2,3]. Another significant phenotypic change of SLD chickens is that the average body weight of homozygous (*dwdw*) chickens was about 40% lower than that of normal chickens [3,4,5]. Considering its advantages in nutrition, management and immunity, the SLD chickens have become a useful tool for chicken breeding [2,3]. The genetic background of the SLD chickens also allowed this strain to become a model system for several human inherited diseases, such as the Laron-type dwarfism, which is characterized by abnormally short stature, obesity, high levels of serum GH and low levels of serum IGF-1 [6,7]. Patients of the Laron syndrome had diverse deletions or point mutations in the *GHR* gene [8,9], and most of these mutations are located in the extracellular domain of the receptor [8]. These mutations caused deficiency in the GH receptor, reduced or abolished the binding affinity of GH to the receptor [7,10], or disrupted the GH signaling, and thereby damaged the normal development of the organism and resulted in dwarfism phenotype. Similarly, the SLD chickens are also caused by diverse deletions or point mutations in the *GHR* gene [2]. However, the precise molecular mechanism of how the *GHR* gene mutations caused so many phenotypic and physiological alterations still remains unclear, and the core regulatory pathways and gene interactional networks underlying dwarfism also need to be further explored.

Skeletal muscle development is a multi-step process that is regulated by diverse growth and transcription factors. IGF-1 plays a critical role in promoting muscle development. IGF-1 null mice die shortly after birth because of severe growth retardation [11], and overexpression of *IGF-1* in skeletal muscle will cause muscle hypertrophy and stimulate muscle cell differentiation [12,13]. In most tissues, the synthesis of IGF-1 is stimulated by GH [14,15], which plays an important role in the regulating somatic growth and development [16]. By binding to the GHR, GH induces the JAK-STAT pathway activation and then triggers IGF-1 synthesis [17]. The reduction of GH/IGF-1 axis is correlated with decreased muscle mass [18], and the activation of the GH/IGF-1 axis can promote both hyperplasia and hypertrophy of skeletal muscle cell [19]. It has been shown that the muscle fiber diameter and the number of muscle fibers are significantly reduced in SLD chickens [20]. The defective *GHR* gene in SLD chickens abolishes the binding and signaling of GH to the GHR, and the GH/IGF-1 axis will be severely damaged accompanying the reduced level of IGF-1, and finally resulting in the reduced muscle cell hyperplasia and hypertrophy. Interestingly, there are multiple signaling pathways that are involved in IGF-1 inducing muscle cell hyperplasia and hypertrophy, such as PI3K-Akt pathway, mTOR pathway and MAPK pathway [21,22]. How do these pathways crosstalk with each other in the skeletal muscle cell of the SLD chicken, and how do the genes in these pathways express and interact in the skeletal muscle cell of the SLD chickens? These questions are greatly significant to further understand the specific mechanism of the defective *GHR* inducing muscle mass loss.

Today, it is understood that microRNAs (miRNAs) play important role in organismal development. miRNA can regulate diverse developmental and molecular processes by controlling gene expression at the post-transcriptional level [23]. Muscle cell is a perfect model system that provides a powerful tool for understanding the signaling systems and transcriptional networks that regulate cell differentiation [24]. Although the transcriptional networks regulating muscle differentiation and growth have been well defined [25], miRNAs as a new layer of regulation provide a new insight into the regulation of muscle biological process. Many miRNAs have been found to be involved in muscle differentiation [26], suggesting their critical role in this process. However, miRNAs expression is dysregulated in cancers and hereditary disease [27,28,29], and their expression profiles are significantly changed during differentiation of muscle cell [30]. Therefore, the expression of miRNAs can be a potential biomarker to monitor the status of cells, and understanding miRNA expression in cellular process or pathological cells is important for exploring the regulatory mechanism or pathogenesis of different cell status.

Recently, we used mRNA and miRNA microarray to test their expression profiles in skeletal muscle of the SLD and normal chickens at embryo day 14 (E14) and seven weeks (7w) of age [31]. From the expression profiles and validation assays we found that let-7b may be involved in higher *GHR* mRNA expression levels in the SLD chickens than in normal chickens, and that let-7b may function in the regulation of many signaling pathways through its direct target gene *GHR* [31]. However, the main difference of gene expression in skeletal muscle between the SLD and normal chickens is still unclear, and the functions and potential interaction of these differential expression genes (DEGs) and miRNAs (DEMs) also remain to be illustrated. In this study, we reused these microarray data to conduct an integrative analysis of mRNAs and miRNA expression profiles and to identify the specific molecular pathways and gene regulation networks in the skeletal muscle of the SLD chickens. The genes and miRNAs involved in normal skeletal muscle development are also included in the integrative analysis. These analyses identified that let-7b, miR-128 and the MAPK pathway are important for chicken muscle development, and their different expressions or activities during muscle development might lead to differences in muscle performance between the SLD and normal chickens. Additionally, we also found some differentially expressed genes and miRNAs between E14 and 7w are critical for skeletal muscle development in both SLD and normal chickens. A regulatory network containing these genes and miRNAs was constructed to show their potential interaction during chicken skeletal muscle development. These findings provide new insights into the molecular regulation mechanisms underlying muscle mass loss in the SLD chickens, and that some regulatory networks are crucial for skeletal muscle development in chickens.

## 2. Results

### 2.1. Muscle Fiber Characteristics in the SLD and Normal Chicken

**Figure 1:**
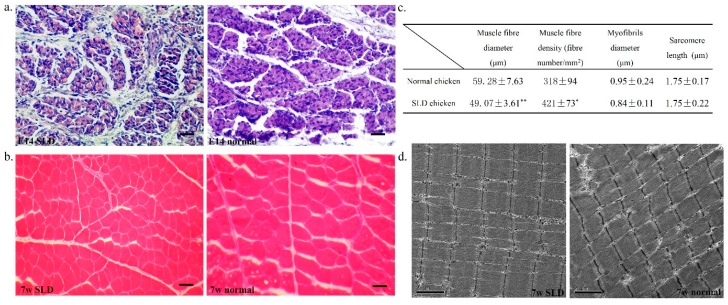
Muscle fiber characteristics in the sex-linked dwarf (SLD) and normal chickens. (**a**) Haematoxylin and eosin (H&E) staining of the leg muscle cross-section from embryo day 14 (E14) SLD (**left**) and normal (**right**) chickens. Bar, 50 µm; (**b**) H&E staining of the leg muscle cross-section from seven weeks (7w) SLD (**left**) and normal (**right**) chickens. Bar, 50 µm; (**c**) Muscle fiber diameter, muscle fiber density, myofibrils diameter and sarcomere length in 7w dwarf and normal chickens. Statistical significance between groups was analyzed by one-sample *t* tests. * *p* < 0.05; ** *p* < 0.01; (**d**) Electron micrograph of a leg muscle cross-section from 7w SLD (**left**) and normal (**right**) chickens. Bar, 2 µm.

To better understand the different performance of skeletal muscle fibers between SLD and normal chickens, we used the muscle section following haematoxylin and eosin (H&E) staining to observe and to analyze the morphology of the muscle fibers. At the E14 stage, developmental muscle fibers in both SLD and normal chickens are unclear and incomplete (Figure 1a), but the muscle fibers in normal chickens are more compact and plumper than the muscle fibers in the SLD chickens. Considering that our previous data have shown that the leg muscle weight in E14 normal chickens is heavier than that in the SLD chickens, it is possible that the skeletal muscle development of these two strains has become different from the embryonic stage on. At the 7w stage, the muscle fibers have developed completely in both of the two strains (Figure 1b). However, it is clear that the muscle fiber diameter in the SLD chickens is smaller than that in normal chickens. Statistical analysis confirmed that the SLD chickens have smaller muscle fiber diameter and higher muscle fiber density than the normal chickens (Figure 1c). Additionally, electron microscopy was also used to assess the microstructure of the skeletal muscle in both of the strains (Figure 1d). The Z band of myofibrils in normal chickens are clearer than those in the SLD chickens, and the myofibrils were observed to be more compact and regular in the SLD chickens than in normal chickens. Otherwise, the myofibrils diameter and the length of sarcomere are not statistically different between the two strains.

### 2.2. Differentially Expressed Genes and miRNAs between SLD and Normal Chickens

**Figure 2:**
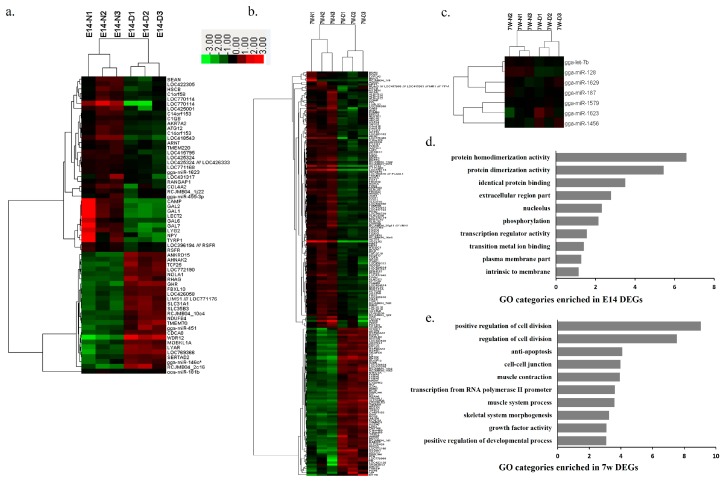
Differentially expressed genes and miRNAs between SLD and normal chickens. (**a**) Hierarchical clustering of 55 DEGs and five DEMs between normal and SLD chickens at E14; (**b**) Hierarchical clustering of 173 DEGs between normal and SLD chickens at 7w; (**c**) Hierarchical clustering of seven DEMs between normal and SLD chickens at 7w; (**d**) GO enrichment for the E14 DEGs. The *y* axis represents GO terms and the *x* axis represents fold enrichment (with *p* value < 0.05); (**e**) GO enrichment for the 7w DEGs. The *y* axis represents GO terms and the *x* axis represents fold enrichment (with *p* value < 0.05).

A total of 55 genes and 173 genes were differentially expressed between the SLD and normal chickens in E14 and 7w, respectively. Only five and seven miRNAs were differentially expressed between the SLD and normal chickens in E14 and 7w, respectively. To visualize the DEGs and DEMs with similar expression profiles across samples, cluster analysis was performed using expression data of the DEGs and DEMs, and Java Treeview were used to show the heat map of the expression of DEGs and DEMs (Figure 2a–c). Furthermore, GO Enrichment Analysis was performed to analyze the functions of these DEGs. At E14 of ages, the functions of DEGs are mainly associated with protein dimerization activity, protein binding and transcriptional regulator activity (Figure 2d). Whereas at 7w of ages, the functions of DEGs are related to cell division, muscle contraction, growth factor activity and muscle system process (Figure 2e), which are implicated in muscle development.

### 2.3. Network and Functional Analysis of the DEGs and DEMs at 7w

**Figure 3:**
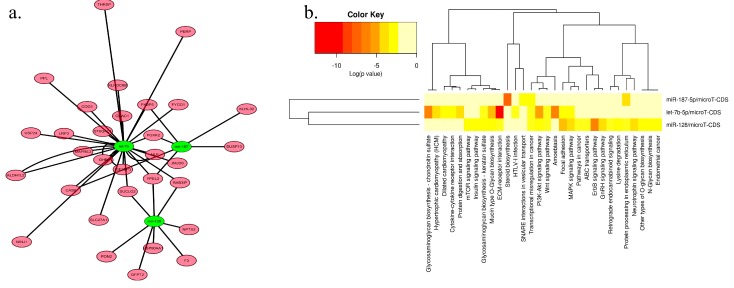

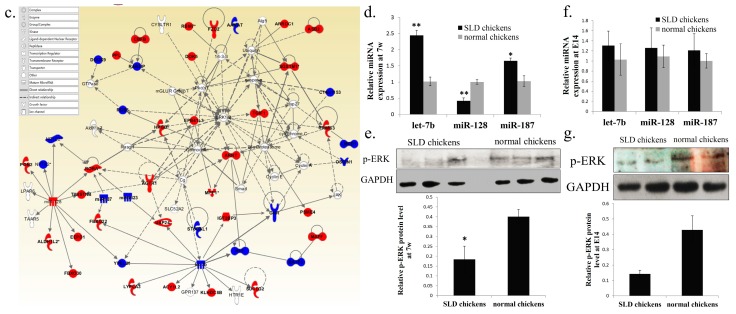
Network and functional analysis of the DEGs and DEMs at 7w. (**a**) Interaction network of DEGs and DEMs at 7w based on MAGIA and Cytoscape software. Green circles represented DEMs and pink circles represented DEGs. Length edges are log *p* value; (**b**) Heat map of putative pathways of DEMs by DIANA miRPath analysis; (**c**) IPA network showing the interaction between DEMs and DEGs at 7w. This network corresponds to cellular development and muscle growth, with ERK1/2 as a hub. Red nodes are the genes or miRNAs that are down-regulated in the SLD chickens at 7w, blue nodes are the genes or miRNAs that are up-regulated in the SLD chickens at 7w; (**d**) qPCR results showed that let-7b, miR-128 and miR-187 are differentially expressed between the SLD chickens and normal chickens at 7w; (**e**) The protein expression of phospho-ERK1/2 (p-ERK) between the SLD chickens and normal chickens at 7w; (**f**) qPCR results of the expressions of let-7b, miR-128 and miR-187 between E14 SLD and normal chickens; (**g**) The protein expression of phospho-ERK1/2 (p-ERK) between E14 SLD (**left**) and normal (**right**) chickens. Results are shown as the mean ± S.E.M. (Standard Error of Mean) of three samples (Figure 3g has only two samples). One sample *t* test was used to assess the different between the SLD chickens and normal chickens. * *p* < 0.05, ** *p* < 0.01.

As the number of DEGs and DEMs at E14 are too few to conduct further analysis, and the functions of the DEGs at 7w are implicated in muscle development, we next focused on analysis the DEGs and DEMs at 7w. By using MAGIA (University of Padua, Padova, Italy) and Cytoscape (University of California, San Diego, CA, USA), we constructed a miRNA-mRNA interaction network at 7w (Figure 3a). Both miRNAs and mRNAs in this network are differentially expressed between SLD and normal chickens at 7w, and the mRNAs in this network are the predicted target genes of the connected miRNAs. This network consists of three nodes for miRNAs, 31 nodes for mRNAs, and 39 edges representing 39 regulations between three miRNAs and 31 mRNAs (Figure 3a). The core nodes for this network are let-7b, miR-128 and miR-187. The length of the edges represented log *p* value of the interaction. To further classify and analyze the potential functions of the three miRNAs in this network, we used DIANA mirPath [32] to investigate the classification and their target pathways. Among these pathways, the PI3K-Akt and MAPK signaling pathways implicated in muscle development were found to be involved in the target pathways of let-7b and miR-128 (Figure 3b and Table S1). Additionally, a functional network identified by IPA (Ingenuity Pathway Analysis) was associated with muscle cell development. This network showed that let-7b and miR-128 are involved in the regulation of many DEMs (Figure 3c). The *ERK* gene, also known as the *MAPK* gene, plays a core role in this network, suggesting that the MAPK signaling pathway may be implicated in this network. Finally, we confirmed that the expressions of let-7b, miR-128 and miR-187 are differentially expressed between the SLD chickens and normal chickens at 7w by using qPCR assays (Figure 3d), and the Western Blotting results also showed that the expression of phospho-ERK1/2 is reduced in the SLD chicken compared to that in normal chickens at 7w (Figure 3e). Additionally, the qPCR results showed that the expressions of let-7b, miR-128 and miR-187 have no significant difference between the SLD chickens and normal chickens at E14 (Figure 3f), but the expression of phospho-ERK1/2 is also reduced in the SLD chickens compared to that of in normal chickens at E14 (Figure 3g). Altogether, these results suggested that let-7b, miR-128 and the MAPK signaling pathway are the most important potential targets that are related to the difference of muscle performance between SLD and normal chickens at 7w of age.

### 2.4. Differentially Expressed Genes and miRNAs between E14 and 7w

**Figure 4:**
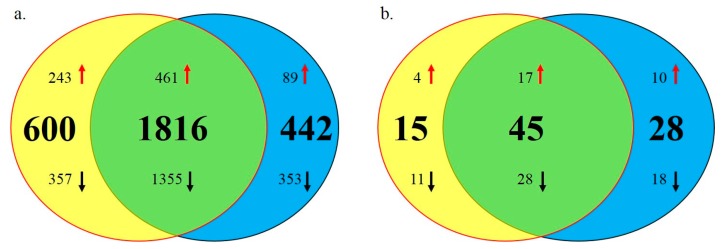
Differentially expressed genes and miRNAs between E14 and 7w. (**a**) Differentially expressed genes between E14 and 7w. The three groups of the Venn diagram represent the normal specific period DEGs (yellow), SLD specific period DEGs (blue), and common period DEGs (green), respectively. The red arrows represent miRNAs that are up-regulated expression between E14 and 7w, and the black arrows represent miRNAs that are down-regulated expression between E14 and 7w; (**b**) differentially expressed miRNAs between E14 and 7w. The three groups of the Venn diagram represent the normal-specific period DEMs (yellow), SLD-specific period DEMs (blue), and common period DEMs (green), respectively. The red arrows represent miRNAs that are up-regulated expression between E14 and 7w, and the black arrows represent miRNAs that are down-regulated expression between E14 and 7w.

The stage from E14 to 7w is an important period of muscle development in chickens. Characteristics of skeletal muscle becomes more and more different between SLD and normal chickens in this period. Therefore, we next analyzed the DEGs and DEMs between E14 and 7w in SLD and normal chickens to investigate the functions of these DEGs and DEMs in skeletal muscle development. In normal chickens, 2416 genes and 60 miRNAs were differentially expressed between E14 and 7w (Table S2). In SLD chickens, 2258 genes and 73 miRNAs were differentially expressed between E14 and 7w (Table S2). Through integrative analysis of these DEGs and DEMs between the SLD and normal chickens, we found that 1816 DEGs and 45 DEMs were commonly differentially expressed in both strains (Figure 4a,b and Table S3). These common DEGs and DEMs may be necessary for the development of chicken skeletal muscle. Further, 600 DEGs and 15 DEMs were specifically differentially expressed in normal chickens (Table S3), while 442 DEGs and 28 DEMs were specifically differentially expressed in SLD chickens (Table S3). These strain-specific DEGs and DEMs may be important for resulting in the different performance of skeletal muscle between the SLD and normal chickens.

### 2.5. Functional and Network Analysis of the Strain-Specific Period DEGs and DEMs

**Figure 5:**
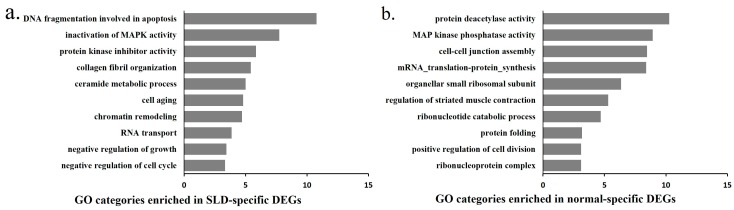

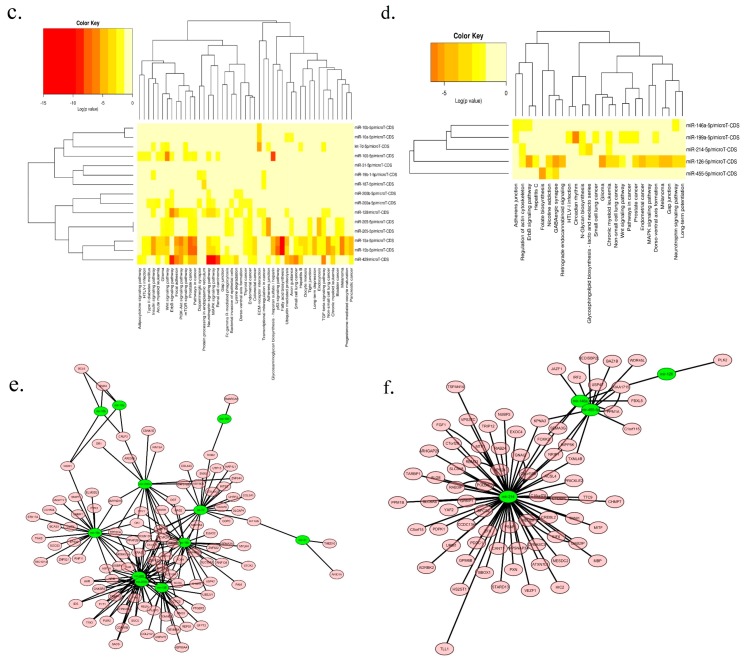
Functional and network analysis of the strain-specific period DEGs and DEMs. (**a**) GO enrichment for the SLD-specific period DEGs. The *y*-axis represents GO terms and the *x*-axis represents fold enrichment (with *p* value < 0.05); (**b**) GO enrichment for the normal-specific period DEGs. The *y*-axis represents GO terms and the *x*-axis represents fold enrichment (with *p* value <0.05); (**c**) Heat map of putative pathways of SLD-specific period DEMs by DIANA miRPath analysis; (**d**) Heat map of putative pathways of normal-specific period DEMs by DIANA miRPath analysis; (**e**) Interaction network of SLD-specific DEGs and DEMs between E14 and 7w based on MAGIA and Cytoscape software. Green circles represent SLD-specific DEMs and pink circles represent SLD-specific DEGs. Length edges are log *p* value; (**f**) Interaction network of normal-specific DEGs and DEMs between E14 and 7w based on MAGIA and Cytoscape software. Green circles represent normal-specific DEMs and pink circles represented normal-specific DEGs. Length edges are log *p* value.

To further understand the functional differences between these strain-specific period DEGs and DEMs, we used GO and mirPath to analyze their functions respectively. The functions of the SLD-specific period DEGs are mainly involved in apoptosis, cell aging, negative regulation of growth and the cell cycle (Figure 5a). However, the functions of the normal-specific period DEGs are mainly involved in protein deacetylase activity, muscle contraction, protein folding and positive regulation of cell division (Figure 5b). For the functions of the DEMs, the SLD-specific period DEMs are enriched in MAPK, PI3K-Akt, mTOR and Insulin signaling pathways, which are related to muscle development (Figure 5c and Table S1), whereas the normal-specific period DEMs are mainly involved in Circadian rhythm, Adherens junction and regulation of actin cytoskeleton (Figure 5d and Table S1). Besides, miRNA-mRNA interaction networks were constructed for the SLD and normal chickens. The network for SLD-specific period DEGs and DEMs consisted of 11 nodes for miRNAs and 103 nodes for mRNAs (Figure 5e), whereas the network for normal-specific period DEGs and DEMs consisted of four nodes for miRNAs and 75 nodes for mRNAs (Figure 5f). Finally, functional IPA networks that are associated with cell proliferation and growth were generated to show the interaction of these strain-specific DEGs and DEMs in chicken skeletal muscle (Figure S1). The results showed that many muscle development-related genes and pathways were integrated into the SLD-specific network, such as *MEF2C*, *MSTN*, *MYH11*, *ACVR2A* and PI3K-Akt pathway, suggesting a specific change of muscle development process in the SLD chickens.

### 2.6. Functional and Network Analysis of the Common DEGs and DEMs between E14 and 7w

**Figure 6:**
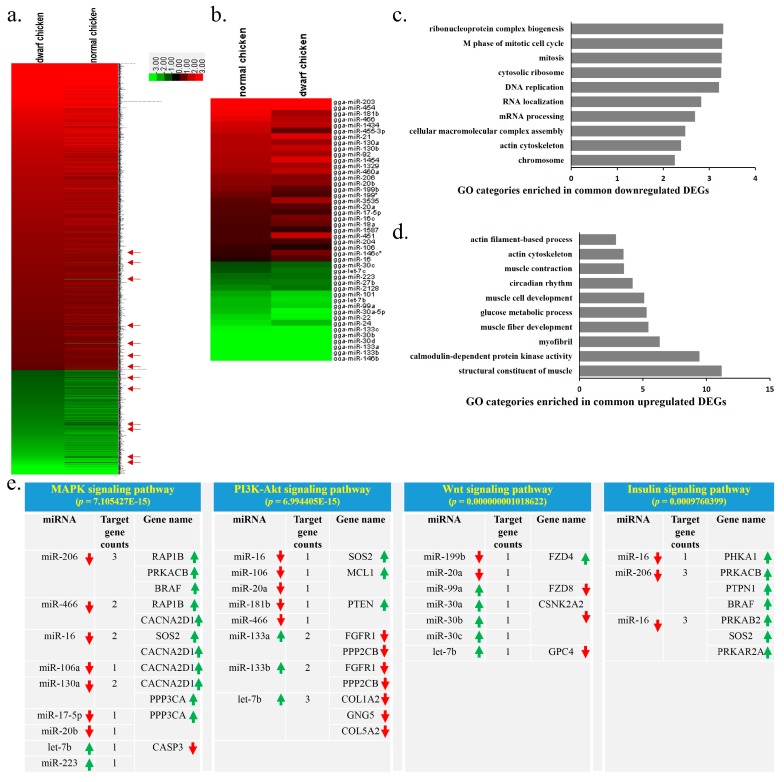

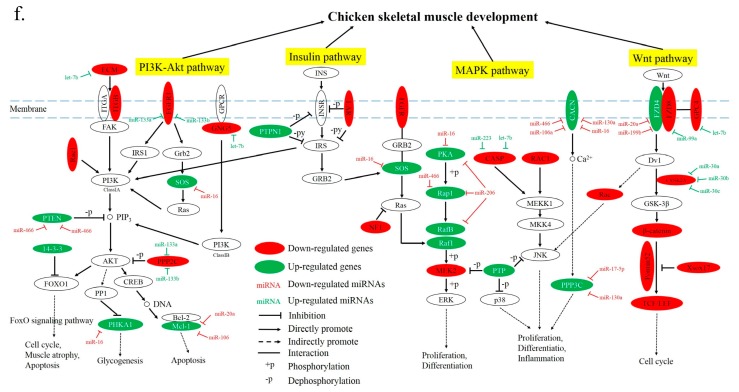
Functional and network analysis of the common DEGs and DEMs between E14 and 7w. (**a**) Heat map of 1355 common down-regulated DEGs (red) and 461 common up-regulated DEGs (green) from E14 to 7w in the SLD and normal chickens. Red arrows indicated 13 DEGs that are displayed different expression trend between SLD and normal chickens; (**b**) Heat map of 28 common down-regulated DEMs (red) and 17 common up-regulated DEGs (green) from E14 to 7w in the SLD and normal chickens; (**c**) GO enrichment for the common down-regulated DEGs. The *y* axis represents GO terms and the *x* axis represents fold enrichment (with *p* value < 0.05); (**d**) GO enrichment for the common up-regulated DEGs. The *y* axis represents GO terms and the *x* axis represents fold enrichment (with *p* value < 0.05); (**e**) List of miRNAs and their target genes that are enriched in the MAPK, PI3K-Akt, Wnt and Insulin signaling pathways. Both of the miRNAs and their target genes are differentially expressed between E14 and 7w. Red arrows indicated down-regulated from E14 to 7w, green arrows indicated up-regulated from E14 to 7w; (**f**) A hypothetical regulatory network showing the interaction of the DEGs and DEMs during chicken skeletal muscle development.

As shown above, there are 1816 genes, and 45 miRNAs are commonly differentially expressed in both SLD and normal chickens between E14 to 7w. Interestingly, among these common DEGs and DEMs, only 13 DEGs displayed different expression trends between SLD and normal chickens (Figure 6a, red arrow). The other 1803 genes and 45 miRNAs show common common up or down regulated expression between SLD and normal chickens (Figure 6a,b). These results suggest that these common DEGs and DEMs play similar and necessary roles in SLD and normal chickens. Therefore, to better understand their roles and relevance in muscle development, we next performed functional and network analysis of these common DEGs and DEMs. The functions of the common down-regulated DEGs are mainly involved in cell cycle, mitosis and DNA replication (Figure 6c), whereas the functions of the common up-regulated DEGs are mainly involved in structural constituent of muscle, myofibril, muscle fiber and muscle cell development (Figure 6d). Next, the potential target pathways of the DEMs were also predicted by mirPath (Table S1). By integrative analysis of these potential target pathways with the DEGs, we found that many DEMs-DEGs pairs which display reverse expression patterns are enriched in the muscle development-related pathways, such as MAPK, PI3K-Akt, Wnt and insulin-signaling pathway (Figure 6e), suggesting that the functions of these DEGs and DEMs are mainly involved in skeletal muscle development. A key network of DEGs-DEMs interactions was constructed by MAGIA analysis and Cytoscape (Figure S2). This network consisted of 15 nodes for miRNAs and 176 nodes for mRNAs. Approximately 15.34% of the DEGs are targeted by at least two DEMs, and the *LASP1* gene is regulated by seven DEMs (miR-24, miR-133a, miR-133b, let-7b, miR-20a, miR-20b and miR-130b). Finally, a hypothetical regulatory network was constructed to show the interaction of the DEGs and DEMs during chicken skeletal muscle development (Figure 6f). This network includes key pathways such as MAPK, PI3K-Akt, Wnt and insulin-signaling pathways, and shows how these pathways cross-talk with each other during muscle development. The key target pairs of miRNAs-mRNAs were also included in this network. Taken together, these results revealed some key pathways and networks of DEGs-DEMs interaction during muscle development, suggesting that the functions of these common DEGs and DEMs during this period are mainly involved in skeletal muscle development.

## 3. Discussion

In the present study, we evaluated and analyzed the difference of the expression profiles of miRNAs and mRNAs during skeletal muscle development between the SLD and normal chickens, and found that some key pathways and networks are implicated in muscle development. SLD chickens deficient in *GHR* are viable models for understanding GH-action and GHR roles at molecular levels [33]. The findings in this study provide direct evidence that *GHR*-deficiency can result in the change of the expression profiles of mRNA and miRNA in chicken skeletal muscle. However, this change may be limited to a certain range, because the number of DEGs and DEMs between SLD and normal chickens are so few, especially at E14 of age. Additionally, by analyzing the DEGs and DEMs between E14 and 7w in SLD and normal chickens, we also found that a small part of genes and miRNAs are specifically differentially expressed in the SLD chickens. This phenomenon was also shown in the *GHR*-mutation type of mice, which exhibited a small portion of genes that were differentially expressed between the *GHR*-mutation and wide type mice [34]. Therefore, to better understand this *GHR*-deficient induced regulatory network in transcriptomes, we constructed several networks by using the DEGs and DEMs between strains and ages to show that the genes and miRNAs are influenced by *GHR*-deficiency, and to describe their potential interactions in skeletal muscle. Finally, by analyzing the functions of these DEGs and DEMs, we also found that several molecules and pathways, such as let-7b, miR-128, and the MAPK signaling pathways, may be the key nodes or pathways that play critical roles in the *GHR*-deficient induced muscle mass loss.

Let-7b, which belongs to the highly conserved *let-7* family, plays a crucial role in development and cell maturation [35]. In our previous study, we found that let-7b inhibits *GHR* expression by directly binding to the 3′ UTR of the *GHR* mRNA [31]. Notably, the binding site of the let-7b in the *GHR* 3′ UTR is located on the deleted region in SLD chickens, therefore, the *GHR* gene expression would not be influenced by the let-7b in the SLD chickens. Additionally, let-7b can also regulate some signaling pathways, which are implicated in cell growth and fat synthesis, by targeting the *GHR* gene [31]. Both our qPCR and microarray results have shown that let-7b is differentially expressed between the normal and SLD chickens at 7w. Functional analysis indicated that many DEGs were targeted by let-7b at 7w, and the potential target pathways of let-7b are mainly involved in muscle development, such as the MAPK pathway and the PI3K-Akt pathway. Up-regulation of let-7b in the SLD chicken might inhibit the normal development of skeletal muscle. Let-7b is able to repress cell proliferation and cell cycle progression [36,37], and our results of functional analysis indicated that many genes in the MAPK or PI3K-Akt pathways might have repressed expression since they are the potential target genes of let-7b. On the other hand, the expression of let-7b is also up-regulated from E14 to 7w in both SLD and normal chickens. Period-specific DEGs potentially targeted by let-7b are involved in the MAPK, PI3K-Akt and Wnt signaling pathways. Therefore, let-7b may be a key regulator in chicken skeletal muscle development.

miR-128 is a brain-enriched miRNA that plays a critical role in nervous system development [38]. In tumor cells, expression of miR-128 is dysregulated with a profound effect on tumorigenesis. Dysregulation of miR-128 can also alter the proliferation, differentiation and metabolism of the tumor cells [39]. Here, we found that miR-128 has lower expression in the SLD chickens skeletal muscle than in normal chickens at 7w, and it is specifically up-regulated from E14 to 7w in the SLD chicken muscle. Network and pathway analysis showed the key roles of miR-128 during the SLD chicken skeletal muscle development. Besides, inhibition of miR-128 reduced differentiation of myogenic satellite cells in turkeys [40], and miR-128 overexpression inhibited proliferation but promoted myotube formation by targeted *myostatin* mRNA in mouse C2C12 myoblast cells [41]. The *myostatin* gene is widely known as a negative regulator of skeletal muscle mass [41]. Reduced expression of miR-128 in the SLD chicken might release the expression of *myostatin* and induce muscle mass loss. Therefore, we suggest that miR-128 may play key roles in skeletal muscle development in the SLD chickens.

The MAPK signaling pathway is a GH-inducible pathway that plays an important role in the development of skeletal muscle [42,43]. GH signaling can lead to the activation of the MAPK pathway through the GH-GHR-JAK2-SHC-MAPK pathway [44]. On the other hand, the MAPK pathway can also be activated by IGF-1 [45], which is a crucial part of GH/IGF-1 axis. Therefore, the MAPK pathway is a potential affected pathway in the SLD chickens. The MAPK pathway is a positive regulator in muscle development [42,46], and also plays a role in the regulation of protein synthesis [47]. During myoblast differentiation, activity of the MAPK pathway would be induced and cooperated with MyoD to activate the transcription of muscle-specific genes [48]. IGF-I-induced muscle hypertrophy would be prevented when the MAPK pathway is inhibited [22]. In the present study, the muscle fiber diameter in the SLD chickens is significantly reduced compared to that in normal chickens, suggesting the reduction of muscle hypertrophy and muscle cell development in the SLD chickens. GO analysis showed that many DEGs in the SLD chicken are implicated in the inactivation of MAPK activity. Functional network analysis and western blot result also showed that the MAPK pathway is a potential key pathway in which there is different activity between the SLD and normal chickens. Therefore, we argue that the activity of the MAPK pathway is a potential influencing factor involved in the *GHR*-deficient induced muscle mass loss. Additionally, functional analysis of the common period DEGs and DEMs also showed the enrichment of these DEGs and DEMs in the MAPK pathway, indicating that the MAPK pathway might play important role in skeletal muscle development of both SLD and normal chickens.

It is generally accepted that the myogenic cells play a key role in skeletal muscle development. Myoblast proliferation and differentiation affects muscle hyperplasia on embryo development days, while the satellite cells are important for myofiber hypertrophy, which is a main factor resulting in the reduction of muscle fiber diameter, after birth [49,50]. The muscle fiber diameter and the number of muscle fibers are significantly reduced in the SLD chickens [20], therefore, the proliferation situation of myogenic cells in this strain might be different from that in normal chickens. On the other hand, GH can promote satellite cell proliferation via its binding to the GHR [51], and the IGF-1 is a critical regulator of satellite cell proliferation and skeletal muscle hypertrophy [51,52]. Therefore, *GHR*-deficiency in SLD chickens would inevitably cause defects in myofiber hypertrophy, as well as in satellite cell proliferation. Our data also support this point. The GO analysis in this study showed that the function of DEGs in the SLD chickens is related to negative regulation of cell cycle and growth, while the function of DEGs in the normal chickens is implicated in positive regulation of cell division and protein synthesis. The functional difference of these DEGs clearly indicate the potential cellular process involved in the *GHR*-deficient muscle mass loss.

Expression of the genes and miRNAs would significantly change from embryo to adult in animals. These transcriptome level changes can result in the phenotypic changes during animal development [53,54]. In this study, we showed that some of the genes and miRNAs are specifically differentially expressed in the SLD and normal chickens between E14 and 7w. These strain specific DEGs and DEMs are important for understanding the molecular mechanism of *GHR*-deficient induced muscle mass loss. The SLD specific DEGs are mainly implicated in inactivation of MAPK activity and negative regulation of growth and cell cycle, whereas the normal specific DEGs are mainly implicated in MAPK phosphatase activity and positive regulation of cell division. As indicated above, the MAPK pathway is important for the development of skeletal muscle [22,42,46,55], and the cell cycle is a crucial process for muscle cell proliferation and differentiation [56,57]. Inactivation of MAPK activity in the SLD chickens might result in the loss of muscle mass. Negative regulation of the cell cycle in the SLD chickens can also inhibit the muscle cell development during E14 to 7w. Additionally, the SLD-specific period DEMs are enriched in the MAPK pathway, suggesting that many genes of the MAPK pathway might be targeted to be inhibited by these DEMs in SLD chickens. Therefore, negative regulation of the cell cycle and inactivation of MAPK activity are two key reasons that determine muscle mass loss in SLD chickens.

Many genes and pathways have been found to be involved in the regulation of skeletal muscle growth from embryo to adult. However, these findings are mainly confirmed in mammals. The core regulators and pathways that influence chicken muscle development from embryo to adult has not been well understood. By analyzing gene expression profiles between broilers and layers across different developmental stages, 543 DEGs and some metabolic pathways have been identified to regulate chicken muscle development [58]. Similarly, the miRNAs and mRNAs transcriptomes of skeletal muscles were also profiled between broilers and layers, and an interaction network of DEMs and their putative targets was constructed to show the potential regulatory network that is involved in the development of chicken muscle [59]. However, these findings just focus on the DEGs or DEMs between broilers and layers. The DEGs or DEMs between different developmental stages haven’t been noticed. In this study, we found a set of common genes and miRNAs that are differentially expressed between E14 and 7w in the SLD and normal chickens, and suggested their potentially critical roles in chicken skeletal muscle development. Therefore, we constructed a regulatory network containing these common DEGs and DEMs to illustrate their potential interactional regulations during muscle development in chickens. Genes among this network are the key players involved in the MAPK, PI3K-Akt, Wnt and Insulin signaling pathways, which are critical pathways for skeletal muscle development. Additionally, the miRNAs among this network also play important roles in skeletal muscle development. miR-206 and miR-133 are two well-known muscle-specific miRNAs that function in the regulation of muscle cell proliferation and differentiation [30,60]. miR-181b and miR-199 are also involved in the regulation of myogenesis [61,62]. Let-7b is a key regulator of development [35], while miR-17-5p, miR-20a and miR-20b are members of miR-17 family, which play important roles during embryo development [63]. Further, miR-16 and miR-223 are involved in muscle cell development in chicken [64]. Therefore, the genes and miRNAs in this network are important for muscle development, and the interaction of miRNAs and mRNAs in this network might provide new insights into the regulation of chicken skeletal muscle development.

Thus, based on the integrative analysis of miRNA-mRNA interactions, we conclude that abnormal expressions of let-7b, miR-128, the MAPK pathway and the negative regulation of cell proliferation may be critical for the muscle mass loss in the SLD chicken. A network constructed with the common period DEGs and DEMs represents a potential regulatory network involved in chicken skeletal muscle development.

## 4. Experimental Section

### 4.1. Histology

H&E staining of the chicken gastrocnemius muscle was carried out as previously described [65]. For determination of ultrastructural morphology, sections of the gastrocnemius muscle were fixed in 3% glutaraldehyde in 0.1 M phosphate buffer (pH 7.4) for 4 h at 4 °C, and subsequently rinsed three times in 0.1 M phosphate buffer. Following rinsing, muscle sections were post-fixed in 1% osmium tetroxide and 1.5% potassium ferrocyanide in 0.1 M cacodylate buffer (pH 7.4) for 60 min. After rinsing three times in phosphate buffer, the samples were then dehydrated and infiltrated with graded mixtures of propylene oxide, finally embedded in Epon. The samples were then examined and photographed using a FEI Tecnai 12 Transmission Electron Microscope (FEI, Eindhoven, The Netherlands). Muscle fiber related parameters were assessed using a JD-801 computer-aided image analyzer (JEDA, Jiangsu, China).

### 4.2. Analyses of Microarray Data

Raw data of the microarrays were processed and analyzed as previously described. |log2FC| ≥ 1, *p* ≤ 0.05 was set as the threshold for selection of differentially expressed gene. |log2FC| ≥ 0.5, *p* ≤ 0.1 was set as the threshold for selection of differentially expressed gene.

### 4.3. Gene Ontology and miRNAs Target Pathway Analyses

Gene ontology enrichment analysis of the DEGs was evaluated by DAVID Bioinformatics Resources 6.7 Functional Annotation tool [66]. For the miRNAs target pathway analyses, DIANA-mirPath tool [67] was used to identify the potential target pathways of the DEMs. As chicken genes were not included in the DIANA-mirPath tool, human miRNAs were used to perform the analysis, and only the miRNAs conserved among vertebrates were used in this analysis. The *p* value threshold in this study was 0.05.

### 4.4. miRNA-mRNA Interaction Analysis

To predict miRNA targets and to analyze the interaction between miRNAs and mRNA, the expression data of mRNAs and miRNAs were integrated using the MAGIA web tool [68], and visualized by Cytoscape [69]. Only the top 250 interactions were used in the visualization of Cytoscape. For the functional network construction, Ingenuity Pathway Analysis [70] was used for analyzing the functional and network connections of the miRNAs and mRNAs. Networks with high enrichment (>30) and are implicated in cell development or muscle growth were selected and merged to show the interaction between the DEGs and DEMs.

### 4.5. miRNAs Quantitative Real-Time PCR (qPCR)

Total RNA from tissues was extracted using RNAiso reagent (Takara, Otsu, Japan). miRNA reverse transcription was perform using Bulge-loop miRNA qRT-PCR Primer Sets (RiboBio, Guangzhou, China) and First-Strand cDNA Synthesis Kit (Fermentas, Burlington, CA, USA) according to the manufacturer’s instructions. The primers specific for gga-let-7b, gga-miR-128, gga-miR-187 and U6 were designed by RiboBio. The qPCR was perform using KAPA SYBR FAST qPCR Kit (KAPA Biosystems, Wobrun, MA, USA) with the annealing temperature of 60 °C for all primers, and quantification was done as previously described [62].

### 4.6. Western Blotting

Western blotting was performed according to the standard procedures. The primary antibodies used in this study were showed as following: anti-phospho-ERK1/2 (Santa Cruz, CA, USA), anti-GAPDH (Bioworld, St. Louis Park, MN, USA). Quantitation of the bands was performed by scanning films using the GBOX-F3 gel documentation system (Syngene, MD, USA), and then the band intensity was evaluated by using Image-Pro plus 6.0 software (Media Cybernetics, Bethesda, MA, USA).

### 4.7. Statistical Analysis

Data are presented as mean ± S.E.M of three replicates. Differences between groups were assessed using one sample *t* test. *p* < 0.05 was considered statistically significant. All experiments were carried out at least three times.

### 4.8. Ethics Standards

Animal experiments were performed in accordance with the regulations and guidelines established by the Animal Care Committee of South China Agricultural University (approval number: SCAU#0015).

## 5. Conclusions

In conclusion, our study revealed that let-7b, miR-128 and the MAPK pathway are potential key molecules and the pathway implicated in *GHR*-deficient induced muscle mass loss, and that the reduced cell division, growth and developmental process in the SLD chicken skeletal muscle may be the molecular reasons that result in the muscle mass loss. Additionally, by analyzing common period DEGs and DEMs between E14 and 7w in the SLD and normal chickens, we found that some DEGs and DEMs are involved in the MAPK, PI3K-Akt, Wnt and Insulin signaling pathways, and play roles in chicken skeletal muscle development. This study provides new insights into understanding the molecular mechanism underlying muscle mass loss in the SLD chickens, and several miRNA-mRNA networks involved in chicken skeletal muscle development.

## Supplementary Materials

Supplementary materials can be found at http://www.mdpi.com/1422-0067/17/3/276/s1.
