# Supplementary material for: Integrative Analyses of miRNA-mRNA Interactions Reveal let-7b, miR-128 and MAPK Pathway Involvement in Muscle Mass Loss in Sex-Linked Dwarf Chickens
Source: Int J Mol Sci. 2016 Feb 24;17(3):276. doi: 10.3390/ijms17030276 (PMC4813140; doi:10.3390/ijms17030276)
Supplement: Supplementary file 1 [file ijms-17-00276-s001.zip › ijms-117079-Supplementary Materials/ijms-117079-supplementary done.pdf]

Supplementary Materials: Integrative Analysis of miRNA-mRNA Interactions Reveals the *let-7b*, miR-128 and the MAPK Pathway to Involve in the Muscle Mass Loss in Sex-Linked Dwarf Chickens

Wen Luo, Shumao Lin, Guihuan Li, Qinghua Nie and Xiquan Zhang

Table S1. Putative target pathways of DEMs by DIANA miRPath analysis.
Table S2. DEGs and DEMs between embryo day 14 (E14) and 7w.
Table S3. DEGs and DEMs that are common or strain-specific expressed between E14 and 7w.
Figure S1. IPA network showing the interaction between SLD-specific or normal-specific DEMs and DEGs.
Figure S2. Interaction network of common DEGs and DEMs between embryo day 14 (E14) and seven weeks (7w) based on MAGIA (University of Padua, Padova, Italy) and Cytoscape software (University of California, San Diego, CA, USA).

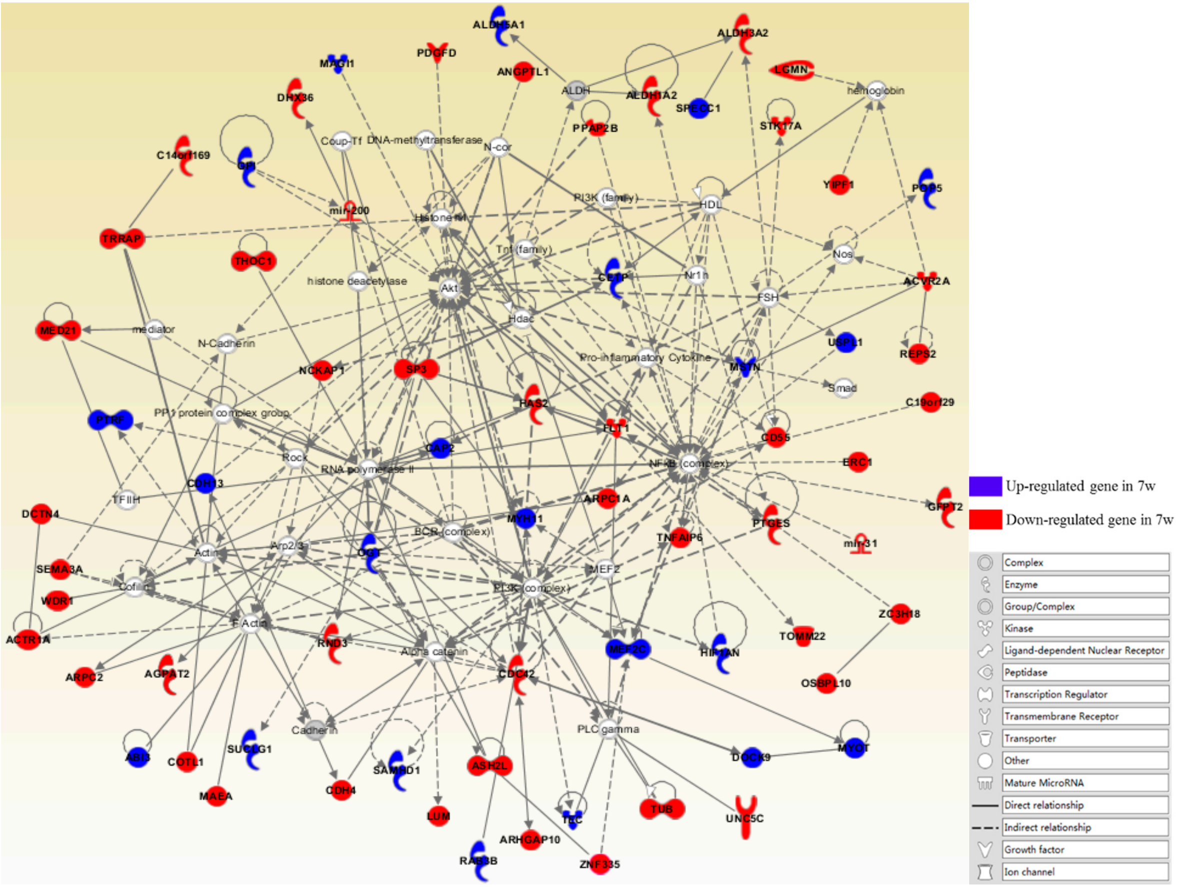

Figure S1. *Cont.*

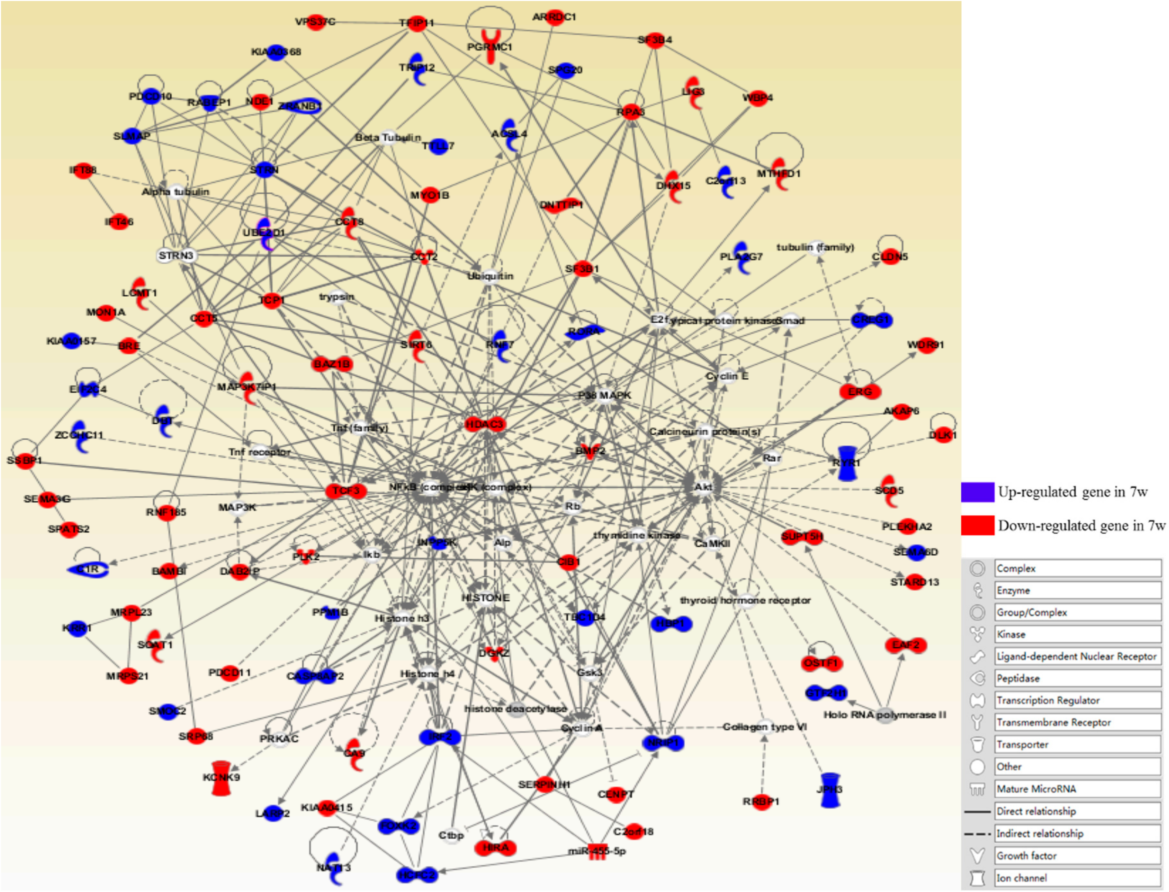

Figure S1. IPA network showing the interaction between SLD-specific DEMs and DEGs.

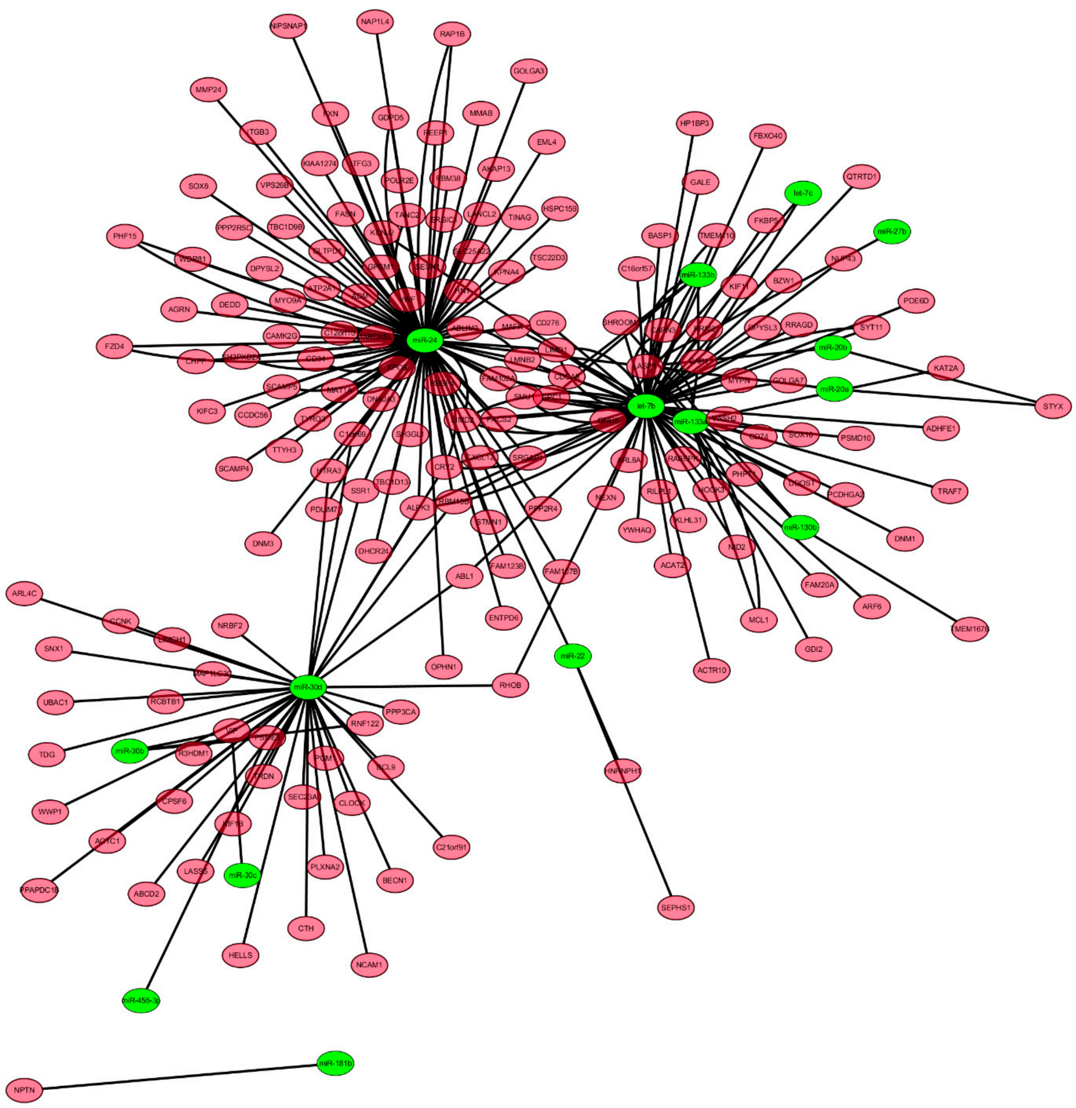

Figure S2. Interaction network of common DEGs and DEMs between E14 and 7w based on MAGIA and Cytoscape software. (Green circles represented common DEMs and pink circles represented common DEGs. Length edges are log *p* value).
